# Compositional tuning of gas-phase synthesized Pd–Cu nanoparticles†

**DOI:** 10.1039/d3na00438d

**Published:** 2023-09-06

**Authors:** Sara M. Franzén, Linnéa Jönsson, Pau Ternero, Monica Kåredal, Axel C. Eriksson, Sara Blomberg, Julia-Maria Hübner, Maria E. Messing

**Affiliations:** a Division of Solid State Physics, Department of Physics, Lund University Lund Sweden sara.franzen@ftf.lth.se; b Occupational and Environmental Medicine, Lund University Lund Sweden; c Ergonomics and Aerosol Technology, Lund University Lund Sweden; d Department of Chemical Engineering, Lund University Lund Sweden; e Carnegie Institution for Science Washington DC USA; f NanoLund, Lund University Lund Sweden

## Abstract

Bimetallic nanoparticles have gained significant attention in catalysis as potential alternatives to expensive catalysts based on noble metals. In this study, we investigate the compositional tuning of Pd–Cu bimetallic nanoparticles using a physical synthesis method called spark ablation. By utilizing pure and alloyed electrodes in different configurations, we demonstrate the ability to tailor the chemical composition of nanoparticles within the range of approximately 80 : 20 at% to 40 : 60 at% (Pd : Cu), measured using X-ray fluorescence (XRF) and transmission electron microscopy energy dispersive X-ray spectroscopy (TEM-EDXS). Time-resolved XRF measurements revealed a shift in composition throughout the ablation process, potentially influenced by material transfer between electrodes. Powder X-ray diffraction confirmed the predominantly fcc phase of the nanoparticles while high-resolution TEM and scanning TEM-EDXS confirmed the mixing of Pd and Cu within individual nanoparticles. X-ray photoelectron and absorption spectroscopy were used to analyze the outermost atomic layers of the nanoparticles, which is highly important for catalytic applications. Such comprehensive analyses offer insights into the formation and structure of bimetallic nanoparticles and pave the way for the development of efficient and affordable catalysts for various applications.

## Abstract 2

Tailored Pd–Cu nanoparticles by spark ablation with a broad compositional range. X-ray spectroscopy and electron microscopy were used to show dynamic synthesis, successful mixing, and insights for development of new catalysts.

## Graphical abstract

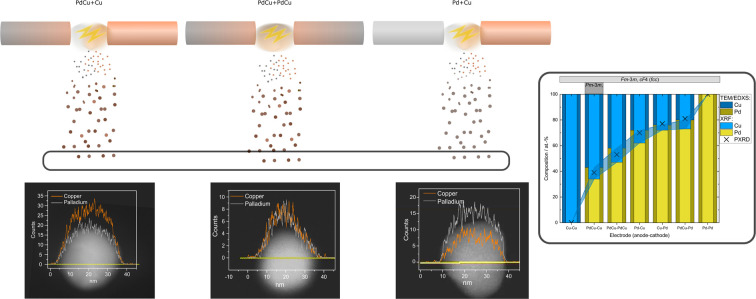

## Introduction

Noble metals, including platinum, palladium, and rhodium, are extensively used for catalysis due to their remarkable capacity to achieve high turnover numbers and exceptional stability during reactions.^1–3^ However, the intrinsic high cost associated with these metals has triggered a need to explore alternative materials that are more abundant, economically viable, while maintaining high catalytic performance. For this purpose, bimetallic nanoparticles emerge as a solution, combining metals to cut costs and fine-tune performance.^4^ For hydrogenation reactions, where focus is directed towards increasing selectivity in an effort to reduce byproducts, palladium-based bimetallic nanoparticles have emerged as exceptional contenders due to their unique electronic configurations and affinity with hydrogen.^5^

Pd–Cu, specifically, can be used for a wide range of catalytic applications such as electrochemical water splitting, chemical sensing,^6^ and C–C cross-coupling reactions,^7,8^ as well as for hydrogenation reactions,^9–13^ for which it has shown exceptional selectivity.^11,14^ The catalytic properties of Pd–Cu nanoparticles are highly sensitive to the chemical composition and need to be optimized for the specific reaction of interest. For example, for CO_2_ hydrogenation to ethanol, Bai *et al.*^9^ found that the highest catalytic activity and selectivity were achieved with Pd-rich Pd_2_Cu nanoparticles, while Wang *et al.*^10^ found that for ethanol electrooxidation, amorphous Pd–Cu was the most efficient. The above examples demonstrate the importance of tailoring the chemical composition of the bimetallic nanoparticles for the reaction that should be catalyzed, although comprehensive understanding of the composition and surface chemistry on the catalytic performance is still needed to achieve full control. Thus, significant attention has been drawn to developing synthesis methods with complete control over the composition of the generated bimetallic nanoparticles.

Traditionally, nanoparticle catalysts are synthesized using chemical methods, such as by reduction or thermal decomposition of metal complexes.^15,16^ These methods have strong limitations for deterministically tuning the properties of the synthesized nanoparticles because of the challenge of isolating the tuning parameters, although a lot of developments have been made in the last decades to synthesize bimetallic nanoparticles with well-defined size and composition.^17–21^ However, the need to use reduction agents and/or stabilizing ligands inherently limits the potential to keep the surface of the nanoparticles free of contamination that might be toxic to the catalyst.^22^ Therefore, physical synthesis methods that both allow for high compositional control as well as contamination-free particle surfaces, are gaining increased interest.

Physical gas-phase methods have the advantage of only using the target material together with an inert gas, minimizing any sources of contaminants. Examples of gas-phase methods that use a solid precursor include thermal evaporation and magneto-sputtering.^23–28^ For thermal evaporation, the solid target is heated in a high-temperature furnace and the generated vapor is consecutively cooled by mixing with a cold gas, resulting in homogeneous nucleation and condensation into nanoparticles. Magneto-sputtering offers a higher versatility in terms of material mixing.^28^ In this method, inert-gas cooling and gas-phase condensation is used for fast quenching of materials into nanoparticles. However, magneto-sputtering faces issues with stability in the production rate. In addition, the vapor is produced by the aid of a plasma which means that a demanding high-vacuum environment is required.

In this work, a physical generation method based on spark ablation is used to generate bimetallic nanoparticles with tuneable chemical composition. Spark ablation is a continuous gas-phase method that enables high purity and high throughput nanoparticles using a simple design. Similar to thermal evaporation, it operates at atmospheric pressures, although, the utilization of high voltage discharges between two easily exchangeable electrodes brings a much higher level of flexibility, compared to thermal evaporation processes. Operations at atmospheric pressure also allow compatibility with common tools for aerosol classification and manipulation, enabling contamination-free generation of nanoparticles of virtually any non-insulating material with a uniform and controllable size distribution.^29–32^ However, there are still aspects of the method that are not completely examined, which are required to employ the full potential of particle mixing with spark ablation.

In the spark ablation method, a vapor is produced from spark discharges between two electrodes connected in an RLC circuit. As a carrier gas transports the vapor from the hot region, it rapidly cools, triggering a supersaturation that leads to nucleation of nanoparticles with the same composition as the vapor.^33^ The nanoparticles grow as they collide and coalesce until reaching a critical diameter at which growth continues by agglomeration to form chains and clusters held together by weak electrostatic forces.^34,35^ The agglomerate particles are compacted into spherical, crystalline, particles in a sintering furnace and size-selected by a differential mobility analyzer (DMA).^36^ Since nanoparticles are generated in the gas phase from electrode feedstocks, the purity of the nanoparticles is only limited by the purity of the gas and of the electrodes.

Bimetallic nanoparticles can be achieved with the spark discharge generator by using electrodes of two different materials and/or alloyed materials.^37–39^ When using electrodes of two different materials, the mass contributions from each electrode will depend both on the material properties of the electrodes and of the circuit properties. Efforts have been made by several groups to gain control over the chemical composition of the bimetallic nanoparticles by changing the electronic parameters of the discharge circuit such as the electrode polarity^39^ and/or the resistance in the RLC circuit.^40^ These methods take advantage of the asymmetry of the current waveform, which increases with higher resistance in the circuit. So far, although being material dependent,^40,41^ the resulting shift in composition has been small. The largest contributing factor to the resulting composition of the bimetallic nanoparticles seems to be the relative ablatability, *i.e.*, the proportion between energy input and produced mass,^31^ of the electrode materials. The ablatability of a material depends on its intrinsic properties, such as melting and boiling temperature, specific heat capacity, melting and boiling enthalpies, and thermal conductivity.^42^ Energy losses during the spark discharges will occur by radiation from the hot spot as light or as heat convection in the surrounding gas, by absorption as latent heat in the electrode material and by conduction along the electrode by metallic thermal conduction.

This work provides further insights into composition optimization and demonstrates the potential of physical synthesis methods for tailoring nanoparticle properties. To understand the mechanisms behind the material contributions to the nanoparticles from electrodes with different chemical compositions, as well as the effect of the electrode polarity, we studied the nanoparticles generated from a set of electrode configurations. As a results, we demonstrate that bimetallic Pd–Cu nanoparticles with a wide range of chemical compositions can be synthesized. Ensemble measurements of the composition were performed using X-ray fluorescent spectroscopy (XRF), which also enabled time-resolved measurements. The composition of single nanoparticles was also determined using transmission electron microscopy (TEM) – energy dispersive X-ray spectroscopy (EDXS) and compositional line scans were obtained using scanning (S)TEM-EDXS to determine the degree of mixing in the nanoparticles. For the application of catalysis, the surface composition is of high importance. Thus, X-ray photoemission spectroscopy (XPS) and X-ray absorption spectroscopy (XAS) measurements were performed with different photon energies to detect any difference in the surface that is not possible to resolve using only STEM-EDXS. ICP-MS measurements were conducted on sintered and agglomerate particles generated by alloyed electrodes as a control measurement. The combination of advanced techniques allows for the comprehensive analysis of the composition, mixing, and surface properties of the bimetallic nanoparticles, providing valuable insights into their formation and structure. The findings pave the way for the development of efficient and sustainable catalysts for various industrial and environmental applications.

## Experimental

### Nanoparticle generation

**Fig. 1:**
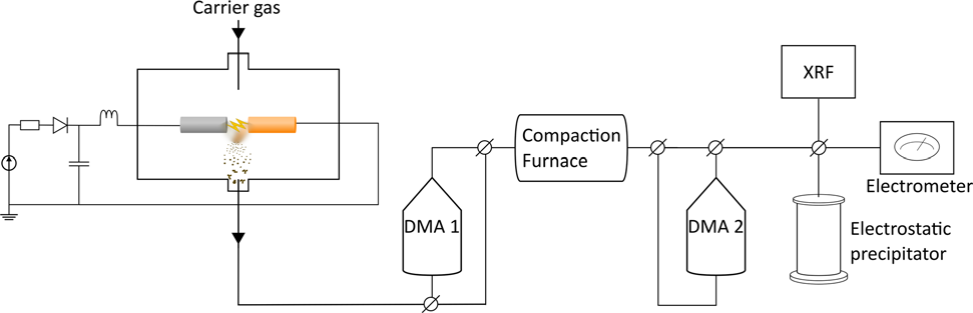
Experimental set-up for the spark discharge generator.

The nanoparticles were generated using a spark discharge generator as described in detail by Ludvigsson *et al.*^43^ A schematic of the setup is shown in Fig. 1. A mixture of nitrogen and hydrogen (95% N_2_ and 5% H_2_) was used as carrier gas at a gas flow rate of 1.68 l min^−1^. Palladium (Pd: Goodfellow, 99.95%, 3.0 mm), copper (Cu: American Elements, 99.9%, 3.0 mm), and palladium–copper alloy (PdCu alloy: American Elements, Cu: 50 at%, Pd: 50 at%, 3.0 mm) were used as electrode feedstocks. The electrode combinations used for this study are listed in ESI (Table SI1†). The electrode distance was 1 mm, the discharge current was 10 mA, the discharge voltage was 2.5 kV, and the discharge frequency was 100 Hz. Two differential mobility analysers (DMA), a standard tool for classifying charged aerosol nanoparticles based on their electrical mobility in a gas stream,^44^ were placed before (DMA 1) and after (DMA 2) the sintering tube furnace. For XRF measurements, ICP-MS, and (HR)TEM/STEM-EDXS the following settings were used: sintered nanoparticles: DMA 1: 55 nm, DMA 2: 30 nm, sintering furnace temperature: 575 °C (Fig. SI1†). Agglomerated particles: DMA 1: 55 nm, DMA 2 and compaction furnace bypassed. For the XRD measurements, the nanoparticles were sintered (at 575 °C) but to achieve a high enough signal, no size selection was performed.

### Characterization

X-ray fluorescence (XRF) spectroscopy was performed on the nanoparticles using an online set-up (Xact 625i), as previously described by Snellman *et al.*^45^ Before the measurements, the aerosol was diluted with N_2_ gas (99.999%) ten times (1.68 → 16.7 l min^−1^). The system sampled on a filter tape for 15 minutes, followed by non-destructive energy dispersive X-ray fluorescence analysis. This enabled time-resolved measurements of the composition of the ensemble of particles measured. XRF measurements were performed for 60 minutes for the different electrode combinations. For the pure electrodes, a shift in composition was observed over this time and measurements were prolonged to 120 minutes for the Cu anode and Pd cathode and to 180 minutes for the Pd anode and Cu cathode.

For HRTEM, TEM-EDXS, and STEM-EDXS the particles were deposited onto Au TEM grids, 300 mesh with a lacey carbon film. The microscope used was a JEOL 3000F. HRTEM images were acquired to find the crystal structure of individual particles. In addition, TEM-EDXS was done on 30 particles per sample to measure the composition of individual particles, as a complement to the ensemble measurement of the nanoparticles with XRF. Line scans and compositional maps were measured on single nanoparticles for each sample.

Powder X-ray diffraction (PXRD) (Stoe Stadi MP, Mythen 1k detector, Cu Kα radiation, *λ* = 1.54178 Å) was employed to determine the crystalline structure of the Pd–Cu samples. For each sample, the particles were collected, bypassing both DMAs to increase the particle concentration and thus enhance the signal-to-noise ratio, onto a Si wafer and later transferred to a Kapton foil to measure in transmission mode. Rietveld refinements were performed with the program Jana2006/Jana2020.^46^ Reflection positions were corrected by using values obtained by measuring and refining PXRD data for a Si standard, as well as samples of elemental Pd and Cu nanoparticles (Pd–Pd and Cu–Cu electrodes) synthesized in the same way as the other samples in this study.

X-ray photoelectric spectroscopy (XPS) and X-ray absorption spectroscopy (XAS) were performed at the FlexPES beamline at the MAX IV laboratory.^47^ A survey scan was done over the energy range 0–1000 eV using a photon energy of 1200 eV. Three different photon energies were used to measure the Cu 2p level (1033 eV, 1233 eV, and 1433 eV) and the Pd 3d_5/2_ level (435 eV, 635 eV, and 835 eV), resulting in a probing depth of about 5.5 Å, 7.5 Å, and 9.5 Å, respectively. For calibration, gold foil was used as a reference at each energy level.

For the inductively coupled plasma mass spectrometry (ICP-MS) measurements NPs were deposited, corresponding to about 40 NPs per μm^2^, onto cellulose filters (MCE, Millipore). The filters and NPs were dissolved in 1 ml aqua regia (HNO_3_/HCl (1 : 3)) for 2 hours at 70 °C. Nine mL of ultrapure water was added and the stock solution was further diluted (10 and 100 times) in 2% HNO_3_ before analysis. Rhodium (Rh), scandium (Sc), and terbium (Tb) were added to each sample as an internal standard. The composition of Pd and Cu was determined by inductively coupled plasma mass spectrometry (ICP-MS, Thermo iCAP Q, Thermo Scientific, Bremen, Germany) equipped with an autosampler (CETAC Autosampler ASX-520). Analysis was performed using the kinetic energy discrimination mode with helium as collision gas. Standards were prepared from 10 ppm multi-element ICP-MS standards (ICP-MS-CAL2-1, ICP-MS-CAL3-R-1, AccuStandard, New Haven, USA). The average levels of the elements in blank filters were subtracted from the samples. Detection limits were 4 ng per sample (^63^Cu) and 7 ng per sample (^105^Pd).

## Results & discussion

**Fig. 2:**
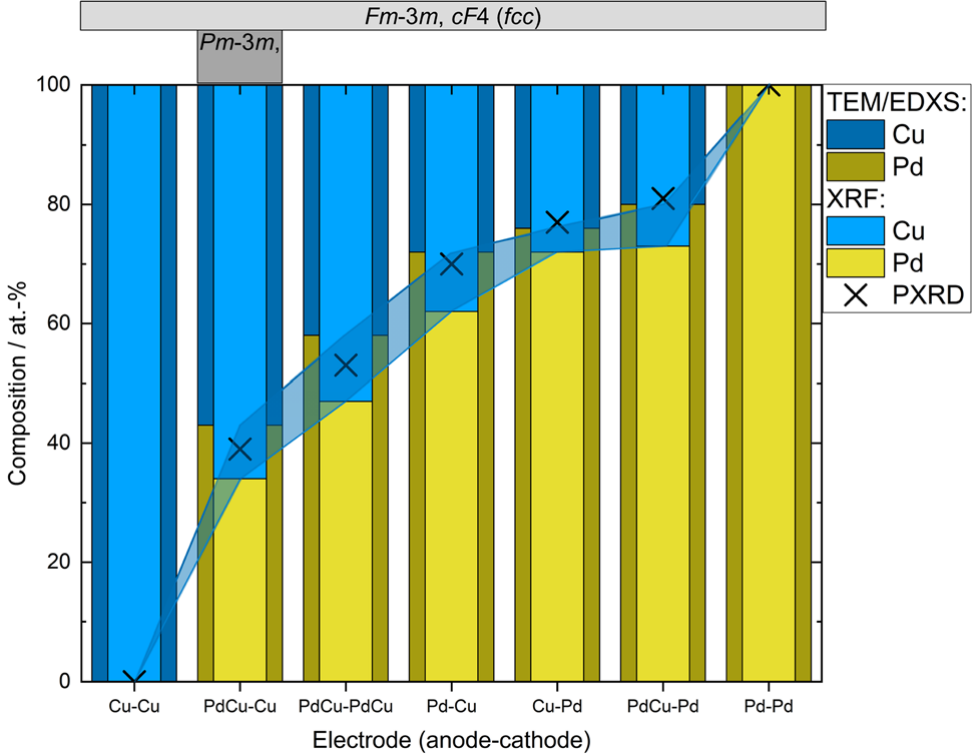
Compositions of nanoparticles generated using different electrode configurations measured by TEM-EDXS and XRF. PXRD compositions were estimated from the linear correlation given by Zen's law (Fig. 3 and Table SI1†). Occurring crystal structure types (fcc or ordered bcc (CsCl-type)) are given in the top panel. Shadowed region marks the difference in the measured composition from the different techniques.

Ensemble measurements of the NP composition was performed by XRF for a large set of electrode configurations. The achieved composition ranged from about 80 : 20 at% to about 40 : 60 at% (Pd : Cu), see Fig. 2. For the alloyed electrodes, for which the elements are already mixed in the electrodes, the elements are ablated with a ratio close to what as is found in the electrodes,^32,34,45,48^ which was also seen here. This is because the amount of evaporated mass during a spark discharge primarily depends on the macroscopic properties of the electrode material such as heat conduction and heat capacity.^31^ Thus, the two metals will have a similar evaporation rate, weighed to the composition of the alloy. The composition of bimetallic NPs generated using electrodes of different materials depends on the composition of the electrodes used, on the ablatability of the material, *i.e.*, the proportionality constant between the energy input and the ablated mass which is determined by the intrinsic material properties,^31^ and on the how much of the spark energy that goes towards ablating material. There have been several attempts to develop predictive models for these factors, although the dependencies are still not completely understood.^37,40^ One well-known factor that contributes to the NP composition when using electrodes of different materials is the polarity. According to Kohut *et al.*,^40^ the shift in composition due to polarity depends on the resistance, which can be used for tuning the NP composition. From the XRF results in this work, the bias due to the polarity is estimated to 45 : 55 (anode : cathode) by calculating the average ablated material from the anode and the cathode when switching the polarity of pure Pd and Cu electrodes. Nevertheless, the Pd electrode made a greater contribution to the nanoparticle composition, regardless of the polarity, and the average composition from the two configurations with pure electrodes was 67 : 33 (Pd : Cu). This is due to the significantly higher ablatability of Pd compared to Cu.^31^ The contributing material from one alloyed and one pure electrode can also be estimated based on the composition of the generated NPs, see ESI and Table SI2.† For the configuration with a PdCu anode and a Pd cathode the material contributions from the electrodes are almost the same. Considering the dominance of the cathode ablation, this indicates that the PdCu alloy has a higher ablatability than pure Pd. This is supported by the higher anode contribution when the PdCu anode is used with a Cu cathode, compared to when the anode is replaced by pure Pd. The higher ablatability of PdCu compared to Pd can be explained by the higher thermal resistivity of PdCu compared to the pure counterparts,^49^ increasing the fraction of the energy that goes towards ablating material.

**Fig. 3:**
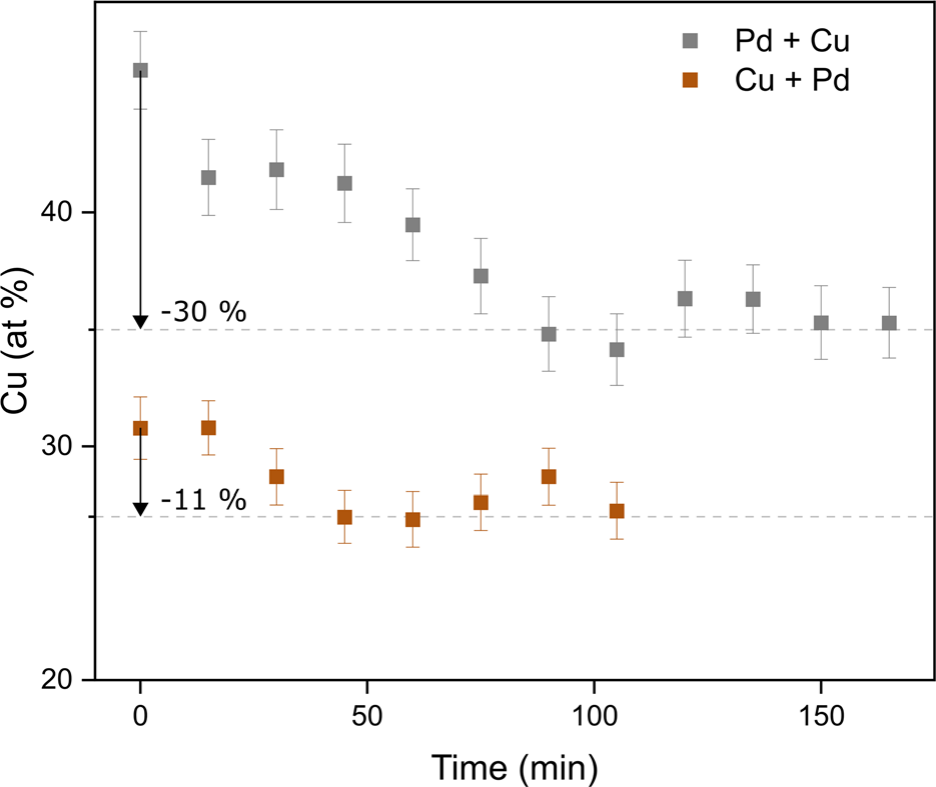
Time-resolved XRF measurements of the composition of the nanoparticles as generated from a Pd anode and a Cu cathode (“Pd + Cu”) and from a Cu anode and a Pd cathode (“Cu + Pd”).

In addition to the ensemble measurements, single particle compositional analysis was done by TEM-EDXS for the same electrode configurations. However, the measurements obtained from TEM-EDXS and XRF exhibit a discrepancy in the resulting composition, with TEM-EDXS consistently showing a lower Cu content compared to XRF measurements. It is not uncommon that only one method is used to determine the NP composition when studying bimetallic NPs,^7,50–52^ and when several methods have been used, any discrepancies are often left uncommented.^53,54^ To address this inconsistency, an additional method, ICP-MS, was employed to measure the composition in the case where the nanoparticles were generated with alloyed electrodes. The results from the measurements in this specific case are summarized in Table 1. It is observed that the XRF measurements align closely with the ICP-MS results, although with slightly higher Cu fraction from XRF. Furthermore, the compositions obtained from all electrode configurations were compared to the compositions calculated from the PXRD results when assuming Zen's law.^55^ These results will be discussed in more detail later in the text. The difference between the measured composition from XRF and TEM-EDXS was largest for the case where the nanoparticles have been generated using a Pd anode and a Cu cathode. In addition, the time-resolved measurements by XRF showed that the composition of the nanoparticles generated by a Pd anode and a Cu cathode changed over time, decreasing Cu concentration by about 30% during the first two hours after the initial spark Fig. 3). This shift in nanoparticle composition is most likely due to re-deposits of Pd onto the Cu electrode, which are clearly visible on the electrode surface after deposition (see ESI and Fig. SI2†). Such material transfer between the electrodes is a well-known phenomenon that is caused by the vapor jet reaching the opposing electrode,^39^*i.e.*, by ion transfer from the anode to the cathode. This effect would be most noticeable when using pure electrodes, and, for the configurations studied here, specifically when Pd is used as an anode as there is more ablated mass from the Pd electrode. These re-deposits change the composition of the electrode surface and cause a shift in the composition of the generated nanoparticles. Because of this drift in composition over time, there will be an additional uncertainty in the actual composition of the nanoparticles. When the electrode polarity was switched, the corresponding decrease in Cu concentration was lower. The effect is smaller because the re-deposition is driven by the ion transfer from the positive to the negative electrode and, during the oscillating sparks, the initially cathodic electrode (Pd) will be more eroded than the initially anodic electrode (Cu), diluting the effect of the re-deposition. Another effect that can contribute to a shift in the composition is the emission profile after the spark is initiated, which can be different for different materials.^34^ Ekimoff and Walters^56^ attribute the emission profile to a phase change in the electrodes as current is delivered during the sparks.

**Table 1:** Cu : Pd atomic ratio in bimetallic nanoparticles generated by alloyed electrodes, measured using XRF, TEM-EDXS, and ICP-MS and estimated from PXRD using Zen's law

Method	XRF	TEM-EDXS	ICP-MS	PXRD
Cu : Pd (at%)	53 : 47	42 : 58	51 : 49	47 : 53
St. dev. (at%)	0.6	0.8	0.4	—a

a *R*-Factors for the PXRD refinement are listed in Table SI1.

Powder X-ray diffraction (PXRD) enabled phase identification and crystal structure refinement. Samples produced without size selection were employed for higher particle concentration and, therefore, improved signal-to-noise ratio. All samples, regardless if generated using electrodes consisting of elemental Pd or Cu or Pd–Cu binaries (for starting electrode compositions, see Table SI1†), predominantly contain an fcc phase (space group *Fm*3̄*m*, Pearson symbol cF4). Solely the most Cu-rich sample (Table SI1†) shows about 15% of an additional phase with an ordered CsCl-type arrangement (*Pm*3̄*m*, cP2), as reported for other Cu–Pd nanocrystalline samples obtained by reduction of organic Pd- and Cu-precursors using ionic liquids or annealing of nanoalloys under H_2_ atmosphere.^58,66^

**Fig. 4:**
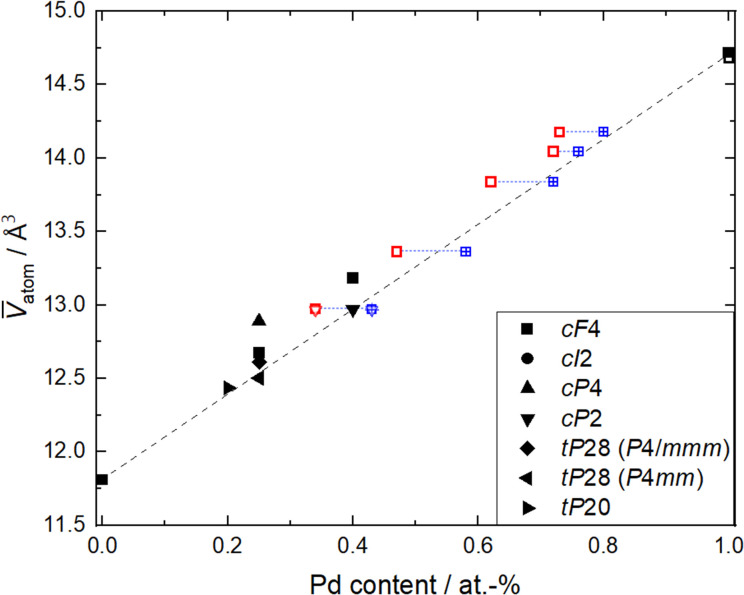
Mean volume *V̄* atom *versus* Pd content for Pd–Cu binaries and elemental Pd and Cu, respectively.^57–66^ Phases are labeled by their Pearson symbol (see Table SI3†). For this study (open symbols), unit cell volume (from Rietveld refinement of PXRD data) was divided by the number of atoms per unit cell and plotted *versus* compositions measured by XRF (red open symbols) and TEM-EDXS (blue open symbols). The dotted blue line connects samples with the same electrode composition. The broken line denotes behavior as expected from Zen's law.

For the purpose of gaining a simple estimate of the variation in the concentration and the unit cell parameters, especially for the disordered alloys observed in the Pd–Cu system, Zen's law was employed. From the refined unit cell parameters of each phase (Table SI1†), the mean volume *V̄* per atom (unit cell volume divided by the number of atoms in the unit cell) was calculated and plotted against the Pd content obtained by TEM-EDXS and XRF (Fig. 2). Known Pd–Cu phases (Table SI3†) were used for comparison. The linear variation of the mean atomic volume (broken line in Fig. 4) with concentration is constructed in accordance with Zen's law for a straightforward comparison of ordered and disordered binaries. Nevertheless, cases in which Vegard's^67^ or Zen's law is strictly obeyed are very scarce.^68^

The mean atomic volumes, in dependence on the Pd content, are both for the samples and the known Pd–Cu binaries (Table SI3†) in good agreement with the linear relation of solid solutions of Cu and Pd according to Zen's law (Fig. 4). As the refinement of Pd/Cu occupancies from the PXRD data was precluded due to the comparatively large peak width observed for these nanocrystalline samples, the composition was estimated from the linear correlation given by Zen's law (Fig. 4 and Table SI1†). For intermediate compositions (up to about 60 at% Pd), the estimated compositions fall in between the values measured by TEM-EDXS and XRF, whereas for higher Pd contents, the estimate tends to agree well with TEM-EDXS data (Fig. 2). The occurrence of an fcc phase as the main component is in sound agreement with the phase diagram,^69,70^ for the applied compaction temperature of 575 °C. For the binary sample with the highest Cu content in this series (Fig. 2), a second phase with an ordered CsCl-type arrangement (*Pm*3̄*m*, cP2) occurs in addition to the main fcc phase. Indeed, a bcc phase is expected below 598 °C (ref. 69 and 70) at this composition, and therefore, this finding denotes a fractional phase transformation due to the particle compaction.

**Fig. 5:**
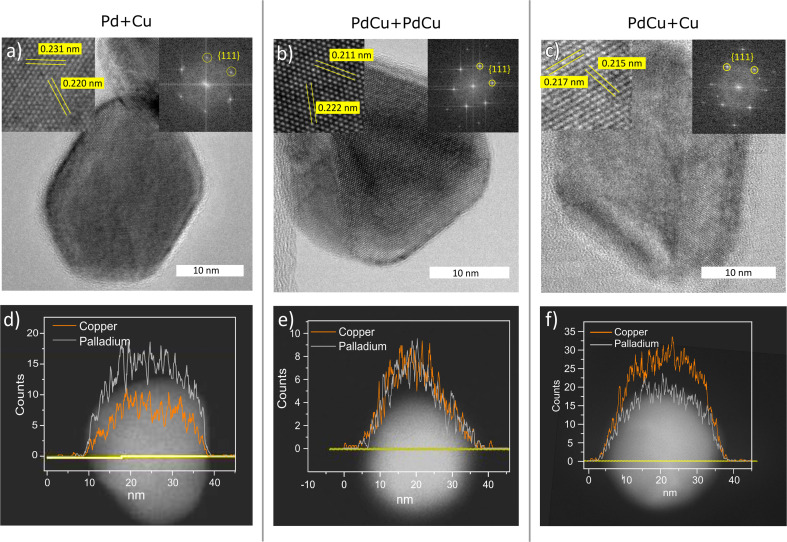
HRTEM (a)–(c) and STEM line scans (d)–(f) of nanoparticles generated with a Pd anode and Cu cathode (a) and (d), PdCu anode and cathode (b) and (e), and a PdCu anode and Cu cathode (c) and (f).

The composition and the phase have been determined on the ensemble level and TEM-EDXS has shown that the standard deviation of the composition in the nanoparticles is low and is thus a good estimate of the composition of the individual nanoparticles. In order to study the distribution of palladium and copper within individual nanoparticles, and also to confirm the crystal structure of individual nanoparticles, STEM line scans and HRTEM were performed. In Fig. 5, the results are summarized for nanoparticles generated with pure Pd and Cu electrodes (Fig. 5a and d), with two alloyed electrodes (Fig. 5b and e), and with one alloyed and one pure Cu electrode (5c and f). From the HRTEM images, we could confirm that the particles had an fcc crystal structure, and from the interplanar spacings, the lattice parameters could be determined. From the STEM line scans, we could see that the palladium and copper were well mixed, regardless of the electrode compositions, which is expected for this material system. However, for catalytic applications, the composition of the outermost atomic layer is extremely important and difficult to study with TEM-EDXS.

**Fig. 6:**
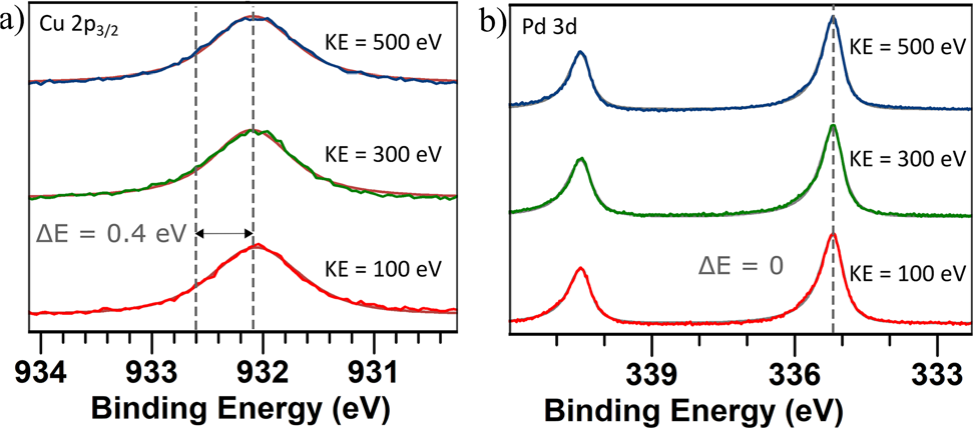
(a) Cu 2p_3/2_ core level spectra and (b) Pd 3d_5/2_ core level spectra of PdCu nanoparticles generated using alloyed electrodes. Three different photon energies were used for each sample to generate photoelectrons with a kinetic energy of 100 eV (red), 300 eV (green), and 500 eV (blue) in order to probe different depths in the samples. The dashed lines show the reported^71,73^ and measured peak values. A Shirley background profile has been applied and subtracted for all spectra.

To determine if there is a shift in composition on the surface, XPS and XAS measurements were performed. A survey scan ranging from 0–1000 eV was performed on the sample with nanoparticles generated using alloyed electrodes. The scan was conducted using a photon energy of 1200 eV (Fig. S4†). From the relative areas of the Cu 2p peaks and the Pd 3d peaks, the composition was calculated to be 44 at% Cu and 56 at% Pd which is close to stoichiometric PdCu. Additional peaks have been identified as originating from Ti which was the support material. The Cu 2p_3/2_ peak was identified at 932.1 eV, which is a shift towards lower energy compared to values reported for metallic Cu^71^ (Fig. 6a). This can be attributed to a charge transfer between the metals.^72^ The Pd 3d_5/2_ was identified at 335.2 eV which corresponds to values reported for metallic Pd^73^ (Fig. 6b). Previous studies of single-crystalline PdCu have also shown a shift in the Cu core levels towards lower energies while the Pd core levels have remained at the same energies.^74^

**Fig. 7:**
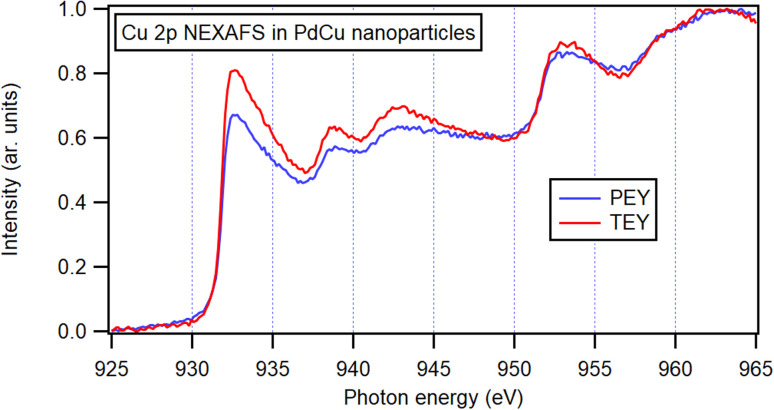
NEXAFS spectra for PdCu nanoparticles generated by alloyed electrodes. Partial electron yield (PEY) and total electron yield (TEY), both normalized to incoming intensity, with pre-edge intensity set to zero and normalized to the maximum intensity.

Since the Cu 2p_3/2_ peak overlaps with that of Cu(i) oxide and thus cannot be identified by only XPS, X-ray absorption spectroscopy (XAS) was performed. Both the total electron yield (TEY) and the more surface-sensitive partial electron yield (PEY) were collected (Fig. 7) and the similarity of the two channels indicating a resemblance between the surrounding of the Cu atoms at the surface and in the bulk of the bimetallic nanoparticles. The spectra exhibit similarities to metallic Cu,^75^ and the observable variations are attributed to the presence of Pd neighbors in the nanoparticles. Furthermore, no obvious signs of oxidation can be identified in the spectra. These observations show that even at shallow atomic layers, there is a resemblance in composition and oxidation state to that of nanoparticles in general.

## Conclusions

We have investigated in depth the chemical composition of nanoparticles generated with different electrode pairs using the ensemble method XRF and measurements of individual nanoparticles using TEM-EDXS. TEM-EDXS measurements show that the deviation of composition between individual nanoparticles is minor. STEM-EDXS line scans over individual nanoparticles also show that the particles are well-mixed. The nanoparticles generated using two alloyed electrodes went through a closer investigation with XPS and XAS which showed that the composition of the outermost atomic layers, of significance for catalysis, was consistent with that deeper into the sample.

We have successfully demonstrated the ability to tune the composition of PdCu nanoparticles using physical methods. By utilizing a small set of electrodes, including pure Pd, Cu, and alloyed PdCu electrodes, we achieved a Pd to Cu ratio of 60–40 to 20–80 at%. Expanding the electrode set to include alloyed electrodes with other compositions would further increase the range of achievable compositions and decrease the step size between them. These findings can be readily applied to other metal binary systems, although adjustments would be necessary to account for the ablatability of the electrode materials. This methodology offers a highly valuable tool for investigating the catalytic dependence on both chemical composition and crystal phase, enabling further exploration in this field.

Time-resolved measurements using XRF showed that the composition of the nanoparticles generated by a Pd anode and a Cu cathode changes over time, a shift that is likely due to re-deposition on the electrode, driven by ion transfer from the positive to the negative electrode. This effect, which illustrated the usefulness of time-resolved composition measurements, can likely be avoided by using pre-alloyed electrodes when possible. However, the phenomenon demonstrates the influence of electrode configuration on nanoparticle composition, and highlights the importance of understanding these factors to accurately determine and control the chemical composition of bimetallic nanoparticle synthesized by physical methods.

## Author contributions

SMF and MEM conceived the project. SMF produced the samples and characterized the materials together with LJ, PT, JMH, MK, and AE. SMF, MEM, JMH, LJ, PT, MK, AE, and SB analyzed the data. SMF wrote the manuscript with contributions from all authors. All authors have given approval to the final version of the manuscript.

## Conflicts of interest

There are no conflicts to declare.

## Supplementary Material

NA-005-D3NA00438D-s001
