# Supplementary material for: Compositional tuning of gas-phase synthesized Pd–Cu nanoparticles
Source: Nanoscale Adv. 2023 Sep 6;5(22):6069–77. doi: 10.1039/d3na00438d (PMC10628985; doi:10.1039/d3na00438d)
Supplement: NA-005-D3NA00438D-s001 [file NA-005-D3NA00438D-s001.pdf]

Electronic Supplementary Material (ESI) for Nanoscale Advances.
This journal is © The Royal Society of Chemistry 2023

Supplementary Information

Compositional tuning of gas-phase synthesized Pd-Cu nanoparticles

Sara M. Franzén,[a, f] Linnéa Jönsson,[a, f] Pau Terneo,[a, f] Monica Kårendal,[b] Axel Eriksson,[c, f] Sara Blomberg,[d] Julia-Maria Hübner,[e] and Maria E. Messing[a, f]

[a] Division of Solid State Physics, Department of Physics, Lund University, Lund, Sweden.
[b] Occupational and Environmental Medicine, Lund University, Lund, Sweden.
[c] Ergonomics and Aerosol Technology, Lund University, Lund, Sweden.
[d] Department of Chemical Engineering, Lund University, Lund, Sweden.
[e] Carnegie Institution for Science, Washington, DC, USA.
[f] NanoLund, Lund University, Lund, Sweden.

E-mail: sara.franzen@ftf.lth.se

Lattice parameters and chemical compositions

Table SI1. Lattice parameters were obtained by PXRD and TEM and compositions were determined by TEM/EDXS and XRF for samples with different electrode materials. Based on the average volume $\bar{V}$

No.	Electrode (anode)	Electrode (cathode)	Lattice parameter PXRD / Å		R_p/ wR_p	Lattice param. TEM / Å	Composition / at.% Pd		
			Fm-3*m*	*Pm*-3*m*		*Fm*-3*m*	TEM/EDXS	XRF	Estimate from Zen's law (PXRD)
611	PdCu	PdCu	3.767(2)		0.0648/ 0.0861	3.75	58	47	53
614	PdCu	Cu	3.730(1)	2.960(1)	0.0725/ 0.0968	3.74	43	34	39
615	Pd	Cu	3.811(4)		0.0332/ 0.0437	3.82	72	62	70
616	Cu	Pd	3.830(1)		0.0140/ 0.0221	3.82	76	72	77
619	PdCu	Pd	3.842(1)		0.0424/ 0.0567	3.87	80	73	81
620	Pd	Pd	3.887(1)		0.0490/ 0.0703				100
621	Cu	Cu	3.615(1)		0.0327/ 0.0432				0

(calculated from unit cell dimensions obtained by Rietveld refinement of PXRD data), the composition was estimated from Vegard's law (see Figure 1).

Nanoparticle size distribution

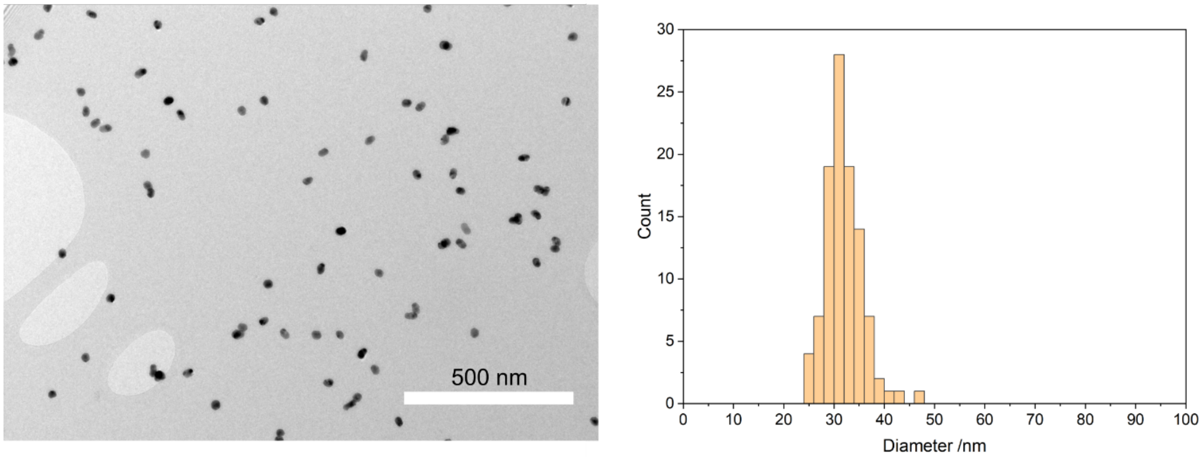

Figure SI1. Low-magnification TEM image of nanoparticles generated by a PdCu anode and a PdCu cathode and histogram of the Feret diameters of the nanoparticles, measured with ImageJ. DMA1: 55 nm, DMA2: 30nm, sintering temperature: 575 ℃.

Relative ablated material

Here we have defined the relative ablated material (in units of moles) of the cathode/anode as $\Delta n_{C/A}/(\Delta n_C + \Delta n_A)$. The ensemble nanoparticle composition from the XRF measurements can, under the assumption that it corresponds to the average composition of the vapor, be used to deduct the relative ablated material from the electrodes. For the configurations with pure electrodes, the relative ablated mass is given directly from the composition as we know that all Pd is provided from the Pd electrode, and all the Cu is provided by the Cu electrode. From the values (given in Table SI2) the average electrode polarity bias can be calculated as the average ablated mass from the anode = 0.45, and from the cathode = 0.55. This indicates that the cathode generates a higher ablated mass, in accordance with what has been previously observed [1]–[3]. We can also get the bias due to the difference in ablatability of Pd and Cu from calculating the average composition from the two configurations, which is 67:33 (Pd:Cu). Using the ablation ratio given by Boeije et al. [4], the composition would be 72:28.

For the configurations with the same materials for the anode and the cathode, it is not possible to deduct anything regarding the relative ablated masses. However, from the configuration with two alloyed PdCu electrodes, we see that the resulting NP composition is 47:53 (Pd:Cu). Assuming that: 1) this composition reflects the ratio of ablated Pd and Cu atoms from the electrodes, 2) this ratio is the same for the anode and the cathode, and 3) this ratio is independent of the circuit properties, the composition of the NPs generated with two alloyed electrodes can be used to calculate the relative ablated material from the configurations with one alloyed and one pure electrode. For example, in the case of a PdCu anode and a Pd cathode, all Cu in the NPs (27 at.%) come from the anode and the Cu generated represents 53 at.% of the total ablated material from the alloyed electrode (from the results

with two alloyed electrodes). Therefore, the relative ablated material from the alloyed electrode should be 0.27/0.53 = 0.51. One concern with using the value ‘0.53’, which is from the atomic percent of Cu in the NPs generated by the alloyed electrodes, is that this value might not be valid when using the alloyed electrode in configuration with another electrode material, e.g., Pd. However, for alloyed electrodes with metals that mix well, the NP composition has been seen to follow closely with the electrode composition, so we do not except significant deviations from this value. A different approach could be to use an average value for the composition from all the different measurement techniques used, or simply to state that this value is close to stoichiometric and use the value ‘0.5’ instead. However, since the rest of the composition values results from the XRF, the authors conclude that the value from the XRF measurement is most suitable also for this purpose. In any case, using either of the other two proposed approaches would generate results that support the conclusion that PdCu has higher ablatability than both Pd and Cu.

The same reasoning can be used to calculate the relative ablated material from the alloyed electrode in the configuration with the Pd cathode. The results are summarized in Table SI2. For the configuration of a PdCu anode and a Pd cathode, it is interesting to see that the relative ablated material from the two electrodes is close to 50:50 and, since there is a bias towards higher ablation from the cathode, this result indicates that the PdCu alloy has higher ablatability compared to Pd. This result is supported when comparing the two configurations with a Cu cathode, the one with a Pd anode, and the one with a PdCu anode. For these configurations, the PdCu anode contributes with a higher relative ablated mass (0.72) compared to the Pd anode (0.62).

Table SI2. Estimate of relative ablated masses based on XRF data on NP composition using different electrode configurations.

Configuration				
Anode	Cathode	NP composition, XRF (Pd:Cu)	$\Delta n_A/(\Delta n_C + \Delta n_A)$	$\Delta n_C/(\Delta n_C + \Delta n_A)$
Pd	Cu	62:38	0.62	0.38
Cu	Pd	72:28	0.28	0.72
PdCu	PdCu	47:53	-	-
PdCu	Pd	73:27	0.27/0.53 = 0.51	0.49
PdCu	Cu	34:66	0.34/0.47 = 0.72	0.28

Re-deposition effects

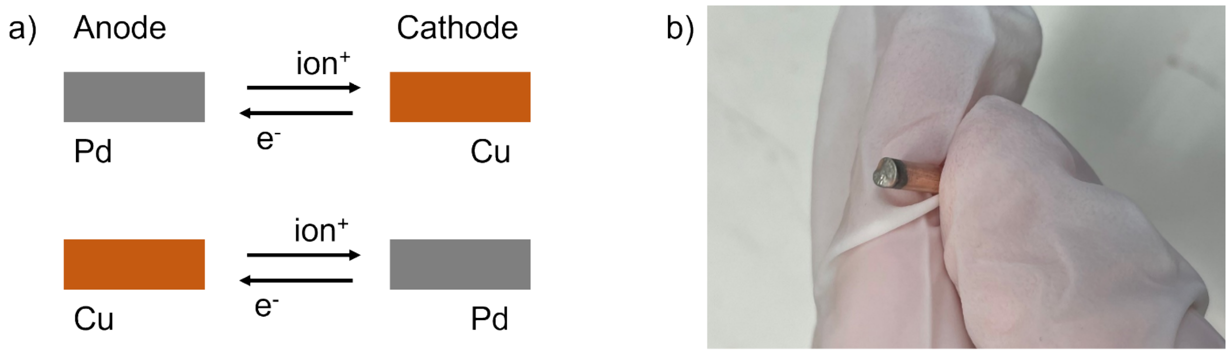

Figure SI2. a) Schematic illustrating the ion and electron exchange during a spark discharge. b) Copper electrode after being used as a cathode together with a palladium anode, showing the re-deposition of palladium.

Re-deposition of material onto the electrodes can be driven by two different effects. One is diffusion and condensation from the vapor cloud that is formed between the electrodes. The other effect is the directed transfer of ions between the electrodes during the spark. In this case, the ions are transferred from the positive to the negative electrode and electrons from the negative to the positive electron (Figure SI2a). The polarity of the electrodes will switch during a spark, but the initially anodic electrode will be positive over longer periods than the initially cathodic electrode, thus, there will be more ion transfer to the initially cathodic electrode. In addition, there will be more material ablated from the material with higher ablatability, which is palladium in this case, so there will be more re-deposition onto the electrode with the lower ablatability.

Powder X-ray diffraction

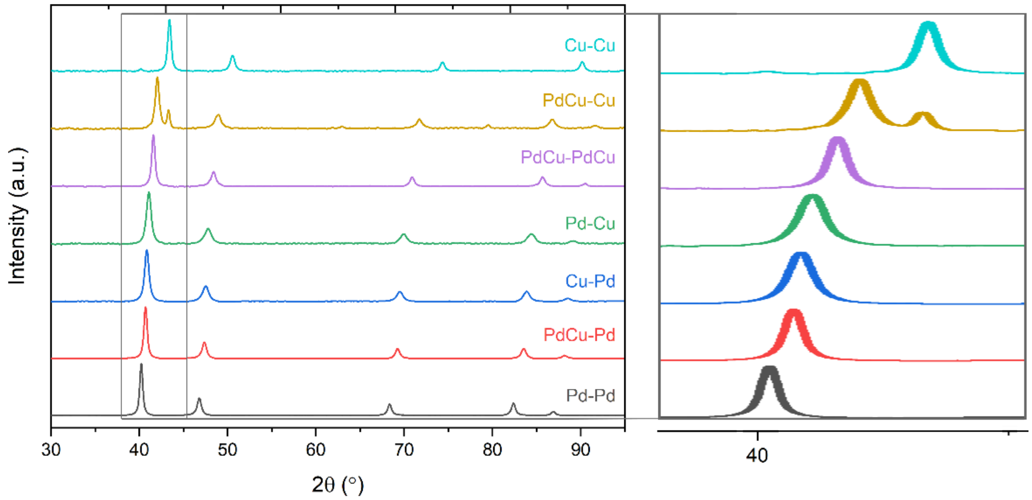

Figure SI3. X-ray diffraction spectra for the bimetallic nanoparticles of different compositions. The measurements were performed on non-size selected nanoparticles.

Table SI3. Space group, Pearson symbol and unit cell dimensions for elemental Pd and Cu, binary Pd-Cu phases.

Composition	Space group, Pearson symbol	Lattice parameter /	Unit cell volume	Reference
Cu	*Fm*-3*m*, *cF*4	3.61491	47.24	Straumanis, M.E.; Yu, L.S. Lattice parameters, densities,

				expansion coefficients and perfection of structure of Cu and Cu-In alpha phase. Acta Crystallographica, Section A: Crystal Physics, Diffraction, Theoretical and General Crystallography (1969) 25, (6) p. 676-682. 10.1107/S0567739469001549
Cu_4Pd	*P*42/m, *tP*20	5.826	248.73	Geisler, A.H.; Newkirk, J.B. Ordering reaction of the Cu4 Pd alloys. Transactions of the American Institute of Mining, Metallurgical and Petroleum Engineers (1954) 200, p. 1076-1082
Cu_3Pd	*Pm*-3*m*, *cP*4	3.722	51.56	10.1088/0370-1301/67/4/303
Cu_3Pd	P4/mmm, tP28	3.710	353.12	10.1143/JPSJ.28.1005
Cu_3Pd	*P4mm, tP*28	3.710	353.12	10.1143/JPSJ.28.1005
$Cu_{0.6}$ $Pd_{0.4}$	*Pm*-3*m*, *cP*2	2.9601	25.94	10.1021/cm902728p
$Cu_{0.6}Pd_{0.4}$	*Fm*-3*m*, *cF*4	3.750	52.73	10.1021/cm902728p
($Cu_{2.85}Pd_{1.15}$)	*Fm*-3*m*, *cF*4	3.701	50.69	Presnyakov, A.A.; Dautova, L.I.; Dzhanbusinov, E.A. The structural forms of the Cu - Pd solid solution of approximate composition Cu3 Pd. Fizika Metallov i Metallovedenie (1963) 16, (1) p. 52-55
CuPd	*Pm*-3*m*, *cP*2	2.987	26.65	10.1039/c0dt01632b
CuPd	*Im*-3*m*, *cI*2	3.063	28.74	10.1039/c0dt01632b
(Cu,Pd)	*Fm*-3*m*, *cF*4	3.766	53.41	Soutter, A.; Colson, A.; Hertz, J. Etude cristallographique des phases ordonees a grande distance et particulierement des structures antiphase monoperiodiques presentes dans les alliages binaires cuivre-palladium. Memoires Scientifiques de la Revue de Metallurgie (1971) 68, (6) p. 575-591
$Cu_{0.134}Pd_{0.866}$	*Fm*-3*m*, *cF*4	3.861	57.56	10.1007/BF00582459
Pd	*Fm*-3*m*, *cF*4	3.8902	58.87	Schroeder, R. H.; Schmitz-Pranghe, N.; Kohlhaas, R.

				Experimentelle Bestimmung der Gitterparameter der Platinmetalle im Temperaturbereich -190 bis 1709 C. Zeitschrift fuer Metallkunde (1972) 63, (1) p. 12-16

High-resolution TEM

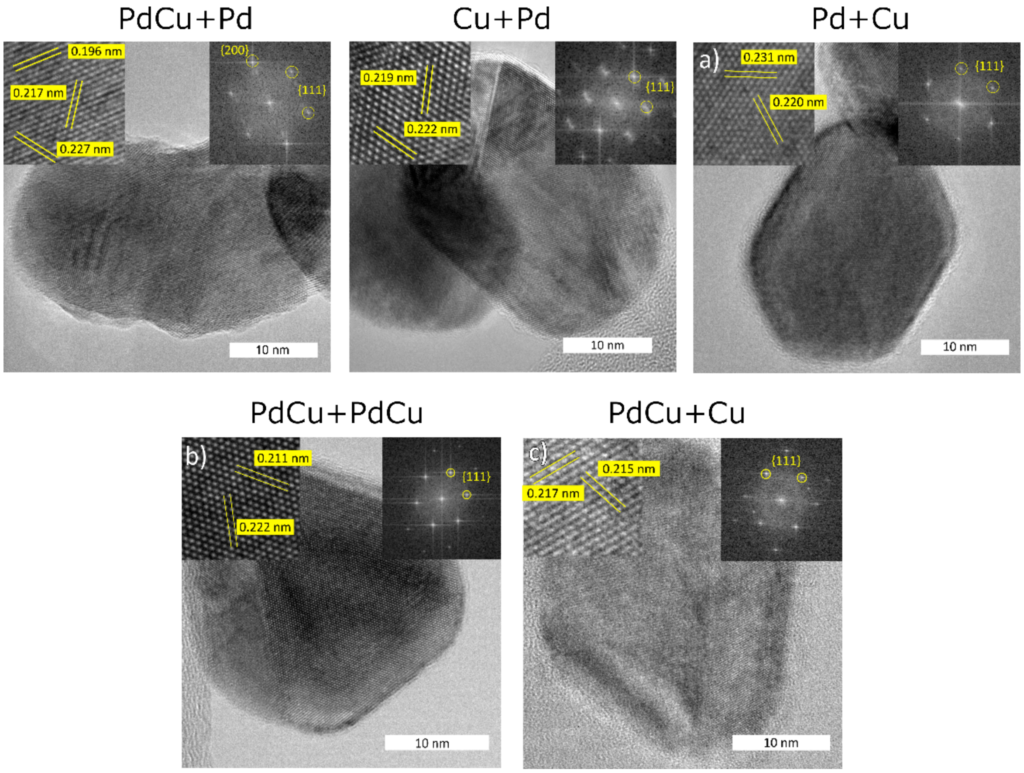

Figure SI3. HRTEM of nanoparticles generated with the different electrode configurations.

XPS survey spectrum

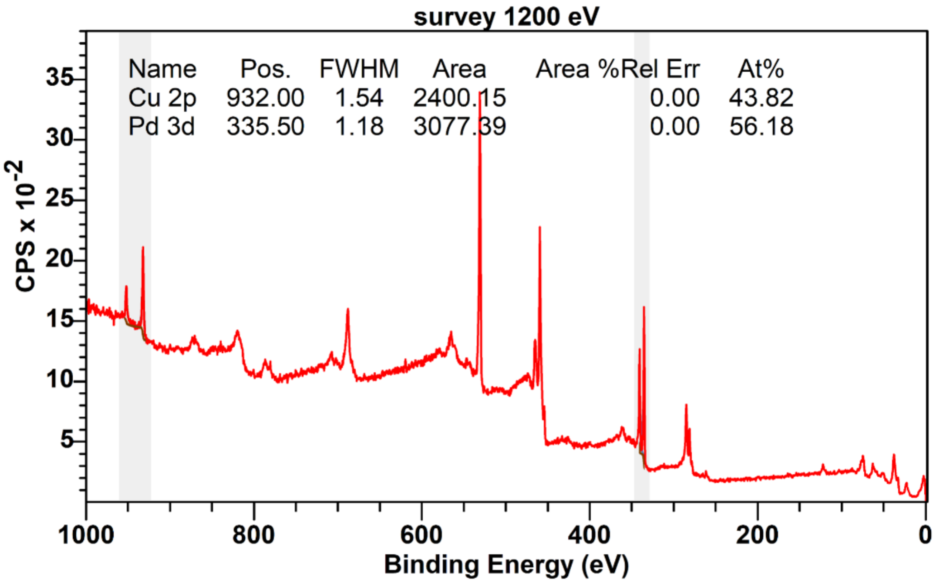

Figure SI4. XPS survey spectrum over binding energies 0-1000 eV using a photon energy of 1200 eV. Composition calculated from the relative peak areas of the Cu 2p and the Pd 3d levels.

[1] N. S. Tabrizi, Q. Xu, N. M. Van Der Pers, U. Lafont, and A. Schmidt-Ott, "Synthesis of mixed metallic nanoparticles by spark discharge," *J. Nanoparticle Res.*, vol. 11, no. 5, pp. 1209–1218, 2009, doi: 10.1007/s11051-008-9568-8.

[2] M. Snellman, P. Samuelsson, A. Eriksson, Z. Li, and K. Deppert, "On-line compositional measurements of AuAg aerosol nanoparticles generated by spark ablation using optical emission spectroscopy," *J. Aerosol Sci.*, vol. 165, no. June, p. 106041, 2022, doi: 10.1016/j.jaerosci.2022.106041.

[3] P. Ternero, M. Sedrpooshan, D. Wahlqvist, and B. O. Meuller, "Effect of the carrier gas on the structure and composition of Co – Ni bimetallic nanoparticles generated by spark ablation," *J. Aerosol Sci.*, vol. 170, no. December 2022, p. 106146, 2023, doi: 10.1016/j.jaerosci.2023.106146.

[4] M. F. J. Boeijes, G. Biskos, B. E. van der Maesen, T. V. Pfeiffer, A. W. van Vugt, B. Zijlstra, and A. Schmidt-Ott (2020). Chapter 2: Nanoparticle Production by Spark Ablation: Principle, Configurations, and Basic Steps toward Application In A. Schmidt-Ott, *Spark Ablation: Building Blocks for Nanotechnology*. Jenny Stanford Publishing Pte. Ltd.
